# Supplementary material for: Friend, not Foe: Lowered Tissue Reactivity to Long-Term Polyimide Implants
Source: bioRxiv. 2026 Feb 9:2026.02.06.703281. Preprint. [Version 1] doi: 10.64898/2026.02.06.703281 (PMC12919039; doi:10.64898/2026.02.06.703281)
Supplement: Supplement 1 [file NIHPP2026.02.06.703281v1-supplement-1.pdf]

bioRxiv preprint doi: https://doi.org/10.64898/2026.02.06.703281; this version posted February 9, 2026. The copyright holder for this preprint (which was not certified by peer review) is the author/funder, who has granted bioRxiv a license to display the preprint in perpetuity. It is made available under aCC-BY 4.0 International license.

Supplementary Figures

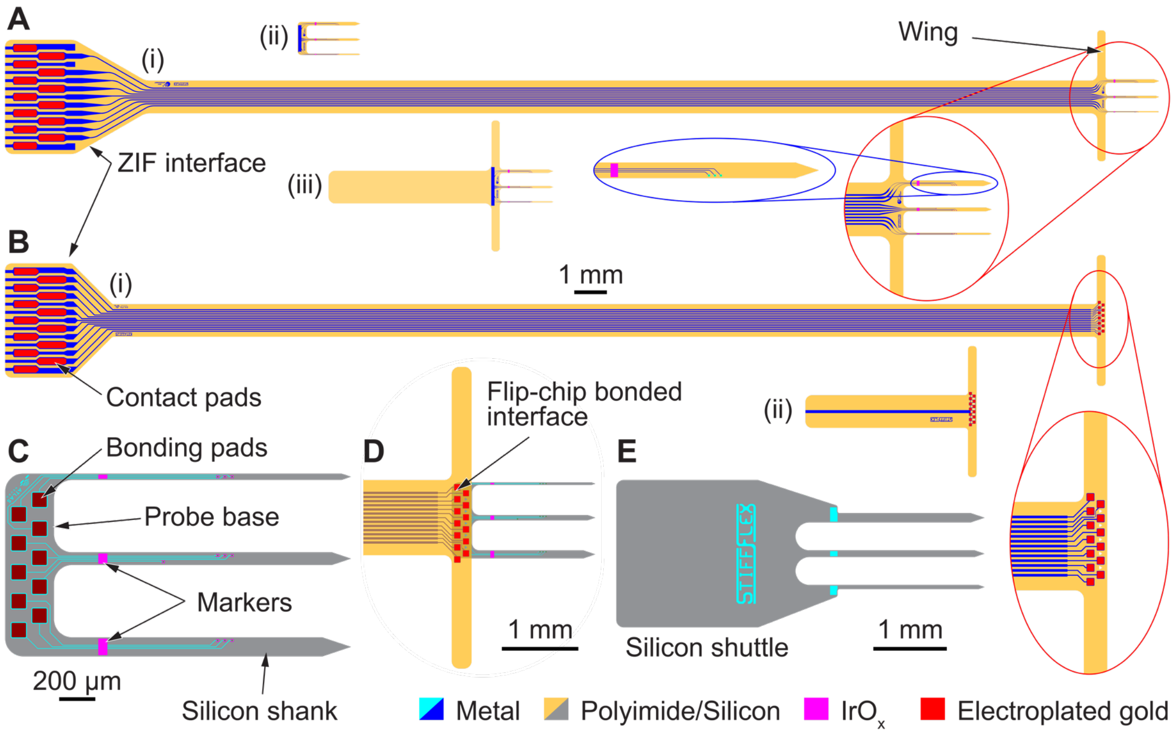

SUPPL. FIGURE 1. Neural probe designs. (**A**) Polyimide probes and test structures with three implantable shanks: (i) Functional probe with a 28-mm-long cable connected to a ZIF interface. The wing structure is used to fix the probe after insertion to the skull with dental cement; (ii) floating implant with a slim base interconnecting the probe shanks, and (iii) tethered implant with a short PI strip mimicking the cable in (i). **(B)** Interconnecting cables for (i) the Si-based probe used for neural recording and (ii) dummy cable with a short PI strip mimicking the cable in (i). **(C)** Silicon probe with 10 bonding pads to which the cable from (B-i) or cable dummy from (B-ii) are flip-chip bonded. **(D)** Flip-chip bonded interface between the Si-based neural probe and the PI-based cable. **(E)** Silicon-based insertion shuttle with a larger base to facilitate the handling of the probe.

bioRxiv preprint doi: https://doi.org/10.64898/2026.02.06.703281; this version posted February 9, 2026. The copyright holder for this preprint (which was not certified by peer review) is the author/funder, who has granted bioRxiv a license to display the preprint in perpetuity. It is made available under aCC-BY 4.0 International license.

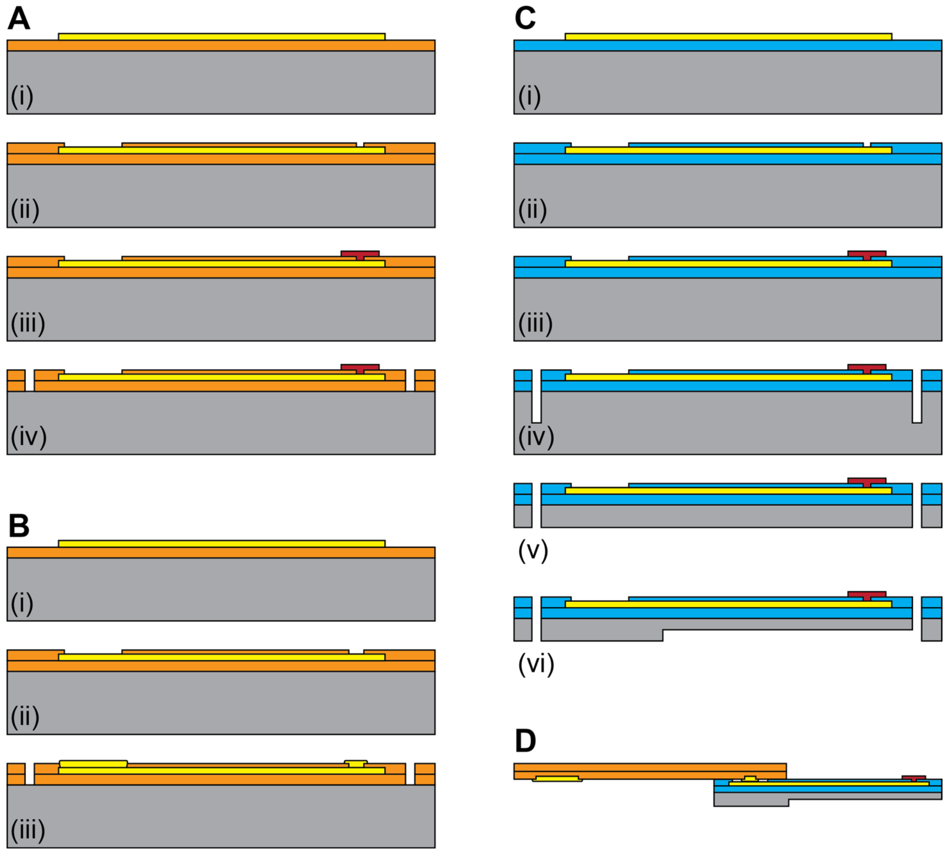

SUPPL. FIGURE 2. Fabrication process. (**A)** Fabrication of PI-based neural probe: (i) Spin-coating of the first polyimide layer, and deposition and patterning of metallization (100 nm Pt) using sputtering and a lift-off process, (ii) spin-coating of the second polyimide layer and RIE to open recording sites (electrodes) and contact pads, (iii) reactive sputter deposition and lift-off patterning of IrO_x electrode coating on top of the polyimide layer, and (iv) patterning of neural probe contour using RIE. **(B)** Fabrication of polyimide interconnecting cable: (i) Spin-coating of first polyimide layer, and deposition and patterning of metallization (100nm Pt) using sputtering and a lift-off process, (ii) spin-coating second polyimide layer and RIE to open contact and bonding pads and definition of the cable contour, and (iii) electroplating of contact and bonding pads with gold. **(C)** Fabrication of silicon probes: (i) Deposition of the first passivation layer containing a layered stack of SiO_x and Si_xN_y using PECVD, and deposition and patterning of metallization (100 nm Pt) using sputtering and a lift-off process, (ii) deposition of second passivation layer stack of SiO_x and Si_xN_y using PECVD and RIE patterning of recording sites and contact pads, (iii) reactive sputter deposition and lift-patterning of IrO_x electrode coating on top of the SiO_x/Si_xN_y passivation layer, (iv) patterning of probe contour by RIE and bulk micromachining of silicon using DRIE, (v) wafer grinding to thin down the silicon substrate, and (vi) selective DRIE to thin down the probe shanks using the EBAG process. **(D)** Silicon probe assembly: Flip-chip bonding of the silicon probe to the polyimide interconnecting cable.

bioRxiv preprint doi: https://doi.org/10.64898/2026.02.06.703281; this version posted February 9, 2026. The copyright holder for this preprint (which was not certified by peer review) is the author/funder, who has granted bioRxiv a license to display the preprint in perpetuity. It is made available under aCC-BY 4.0 International license.

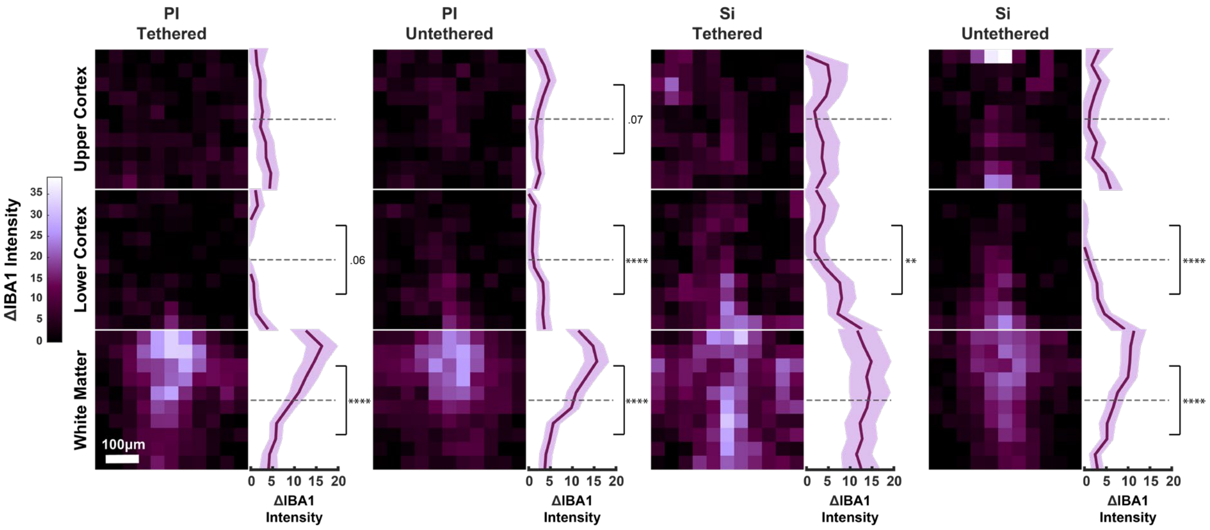

SUPPL. FIGURE 3. Microglial depth profile for the tethered and untethered silicon and polyimide probes. Scale bar = 100 µm. PI = Polyimide; Si = Silicon. Stacked heatmaps correspond to upper cortex (top), lower cortex (middle) and white matter (bottom). Line graphs display the average ΔIBA1 value across cortical depth. Shaded areas represent SEM. Significant differences of signal intensities in upper and lower areas within each ROI are marked. All conditions display an increased ΔIBA1 intensity in the white matter. * $p < .05$, ** $p < .01$, *** $p < .001$, **** $p < .0001$.

bioRxiv preprint doi: https://doi.org/10.64898/2026.02.06.703281; this version posted February 9, 2026. The copyright holder for this preprint (which was not certified by peer review) is the author/funder, who has granted bioRxiv a license to display the preprint in perpetuity. It is made available under aCC-BY 4.0 International license.

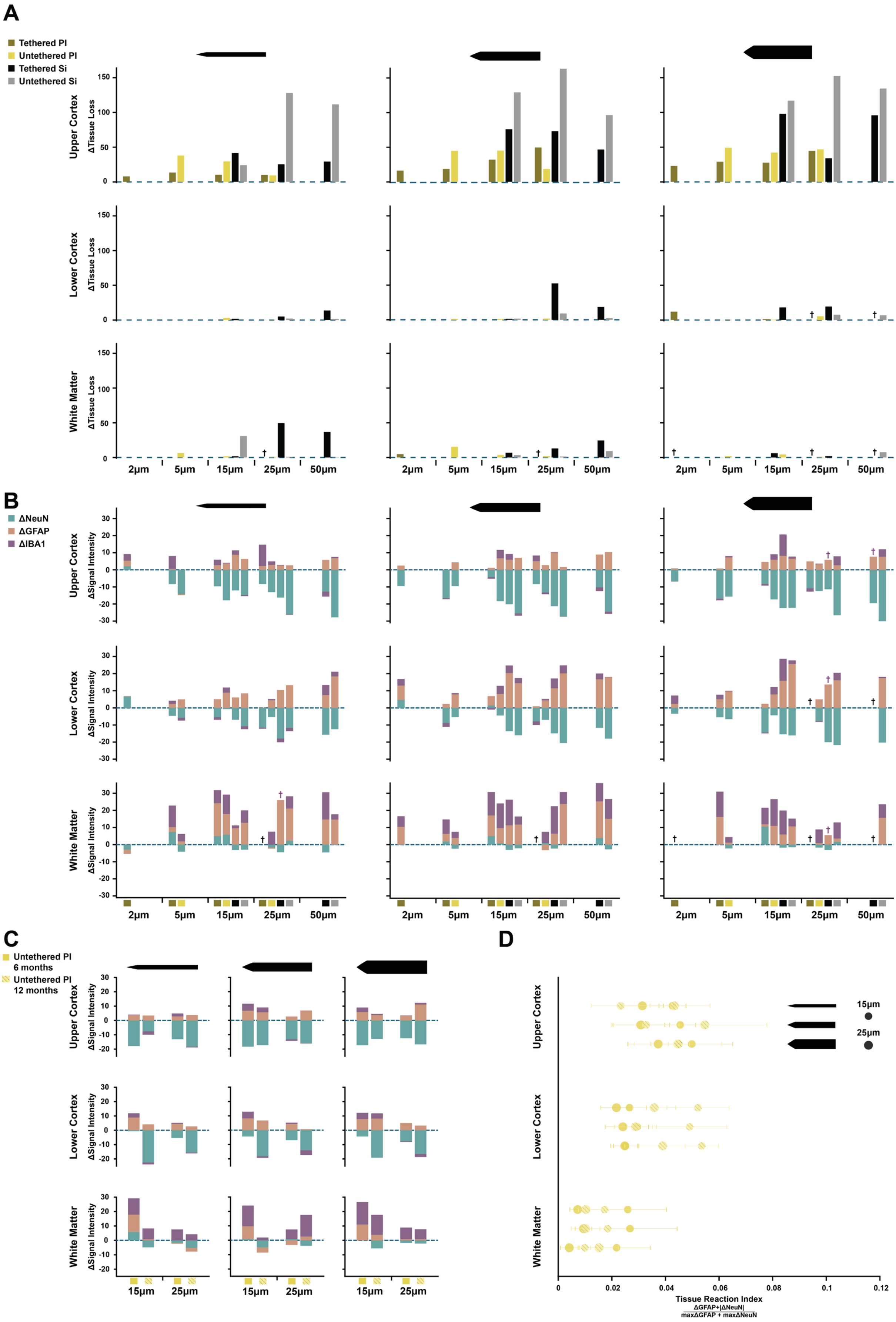

bioRxiv preprint doi: https://doi.org/10.64898/2026.02.06.703281; this version posted February 9, 2026. The copyright holder for this preprint (which was not certified by peer review) is the author/funder, who has granted bioRxiv a license to display the preprint in perpetuity. It is made available under aCC-BY 4.0 International license.

SUPPL. FIGURE 4. Tissue responses across all experimental conditions. **(A)** ΔTissueLoss caused by the various probes with a shank of 35 (left), 70 (middle column), 105 µm width (right) in upper cortex (top), lower cortex (middle row) and white matter (bottom). **(B)** ΔNeuN (neuronal loss), ΔGFAP (astrocytic response) and ΔIBA1 (microglial response), in the same format as panel A. The colors below the x-axis represent the various material and tethering combinations. Black plusses mark missing datapoints for conditions with one sample or less. Colored plusses mark missing datapoints in a specific staining. **(C)** Untethered polyimide probes implanted for 6 or 12 months of shanks with a width of 35 (left), 70 (middle column) and 105 µm shank width (right column). **(D)** $Idx_{TissueReaction}$ for untethered polyimide probes implanted for 6 and 12 months.

bioRxiv preprint doi: https://doi.org/10.64898/2026.02.06.703281; this version posted February 9, 2026. The copyright holder for this preprint (which was not certified by peer review) is the author/funder, who has granted bioRxiv a license to display the preprint in perpetuity. It is made available under aCC-BY 4.0 International license.

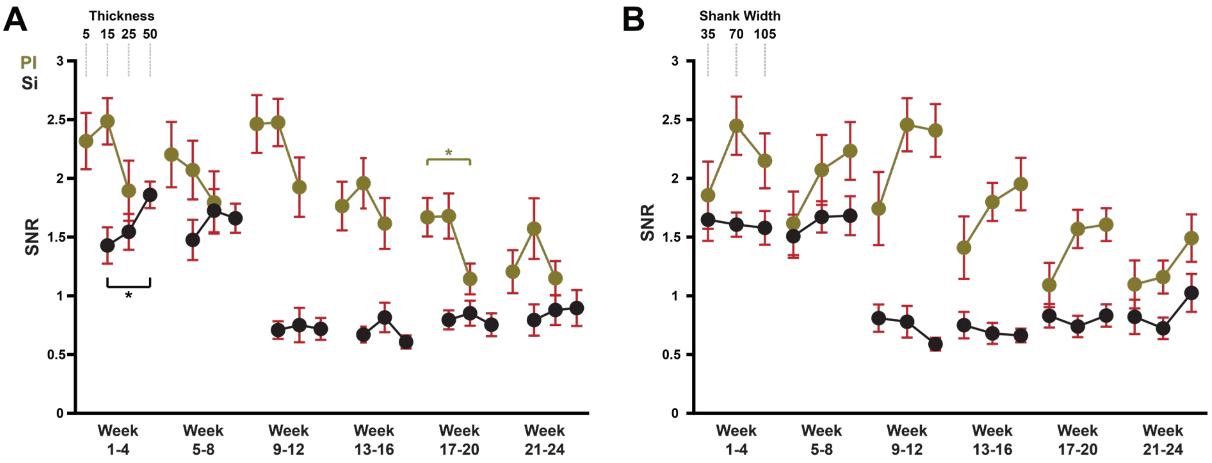

SUPPL. FIGURE 5. Average signal-to-noise (SNR) ratio for electrodes on shanks with different thicknesses and widths in 4 week time-bins. PI = Polyimide; Si = Silicon. Error bars, SEM. **(A)** Influence of probe thickness on SNR. Polyimide probes had a thickness of 5, 15, or 25 µm and silicon probes were 15, 25 or 50 µm thick. **(B)** SNR of electrodes from shanks with a width of 35, 70 or 105 µm. * $p < .05$.

bioRxiv preprint doi: https://doi.org/10.64898/2026.02.06.703281; this version posted February 9, 2026. The copyright holder for this preprint (which was not certified by peer review) is the author/funder, who has granted bioRxiv a license to display the preprint in perpetuity. It is made available under aCC-BY 4.0 International license.

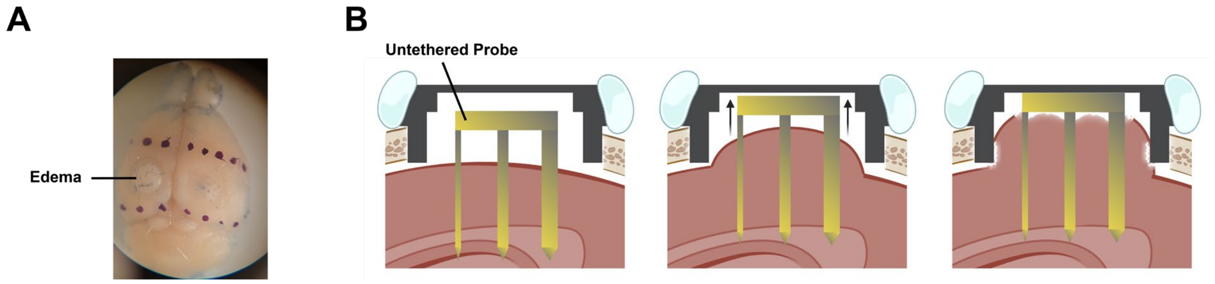

SUPPL. FIGURE 6. Adverse effects of the surgical method used to implant untethered probes. We made a 3 mm circular craniotomy and closed the skull with a stainless-steel cap. **(A)** Example brain with edema at the site of the implantation in the left hemisphere. **(B)** Illustration of the possible negative impact of the cavity above the cortex. Pressure may cause displacement of the cortex and probe into the cavity in the direction of the inner roof of the cap.
